# Emergency Medicine Resident Burnout and Examination Performance

**DOI:** 10.1002/aet2.10527

**Published:** 2020-10-11

**Authors:** Lara Z. Vanyo, Deepi G. Goyal, Ramnik S. Dhaliwal, Randy M. Sorge, Lewis S. Nelson, Michael S. Beeson, Kevin B. Joldersma, Jayram Pai, Earl J. Reisdorff

**Affiliations:** ^1^ Mount Sinai Health System New York NY USA; ^2^ Department of Emergency Medicine Mayo Clinic Rochester MN USA; ^3^ Department of Emergency Medicine Carepoint Health–Swedish Hospital Denver CO USA; ^4^ Department of Emergency Medicine Louisiana Health Science Center New Orleans LA USA; ^5^ Department of Emergency Medicine Rutgers New Jersey Medical School Newark NJ USA; ^6^ Department of Emergency Medicine Summa Health Akron OH USA; ^7^ American Board of Emergency Medicine East Lansing MI USA; ^8^ Department of Emergency Medicine Alpert Medical School Brown University Providence RI USA

## Abstract

**Objectives:**

Burnout afflicts emergency physicians (EPs) to a significant degree. The impact of burnout spans from decreased clinical efficiency to increased medical errors to heightened risk of physician suicide. This large‐scale study captures responses from emergency medicine (EM) residents regarding two burnout items and examines the correlation between in‐training examination (ITE) scores and burnout risk as well as that between residency year and burnout risk.

**Methods:**

This was a prospective, mixed‐methods, cross‐sectional cohort study. All residents in U.S. categorical EM residents who took the 2019 ITE were included. At the end of the ITE, residents were invited to complete a voluntary survey that included two items from the Maslach Burnout Inventory (MBI) that have been found to be strongly indicative of burnout: one about self‐perception of being burned out and one about feelings of callousness. Responses were on a 7‐level Likert scale (1–7), ranging from very low frequency (1) to very high frequency (7). Measurements included the number of residents in each year‐level of training (EM1–EM4), the MBI item ratings, and the ABEM ITE score. Performance, as measured by the scaled, equated score, was compared to the MBI item responses. A corrected Spearman’s correlation coefficient (ρ) was used to compare continuous data (score) against a discrete ordinal variable (MBI Likert response).

**Results:**

There were 2,501 EM1 residents, 2,389 EM2 residents, 2,206 EM3 residents, and 616 EM4 residents in the study group. There were 7,206 (93.4%) physicians who completed the first MBI question about burnout; 7,172 (93%) completed the second MBI question about callousness. There was no statistically significant association between the burnout item response and ITE performance (ρ = –0.03; p = 0.015). There was a positive, statistically significant association between the callousness item response and higher ITE performance (ρ = 0.07; p < 0.001). There was a statistically significant association between the response to the burnout item and training level (ρ = 0.07; p <0.001). There was also a statistically significant association between the response to the callousness item and training level (ρ = 0.15; p < 0.001). The overall prevalence of burnout risk in various training levels were EM1, 28.2%; EM2, 39%; EM3, 41.1%; and EM4, 43.3%.

**Conclusions:**

Our study found no significant correlation between ITE score and burnout risk. There was a weakly positive correlation between ITE scores and callousness. Based on our study results, ITE scores may not be useful in prognosticating burnout risk for EM residents and, interestingly, higher ITE scores correlated to stronger feelings of callousness. Our study indicates that EM residents at higher levels of training reported stronger self‐perceptions of burnout and callousness. Further investigation into why residents at higher levels of training may experience greater burnout risk is warranted.

## Background

Burnout is a complex multifactorial syndrome that affects the majority of physicians at some point in their career. Burnout in emergency physicians (EPs) is among the highest in medicine, with one report showing burnout in emergency medicine (EM) reaching 60%.^1^ In 2019, the National Academy of Medicine published “Taking Action Against Clinician Burnout,” which identified burnout as one of the most concerning and prevalent problems in medicine.^2^ Across specialties, physician burnout has been implicated in higher rates of medical error, decreased empathy, disregard for social impact on illness, and increased racial bias among residents.^3^ On the individual level, burnout has been implicated in poor job satisfaction, increased incidence of drug and alcohol use, and increased thoughts of suicide and self‐harm.^4^, ^5^ Burnout may impact job satisfaction, patient safety outcomes, and career longevity.

Defining burnout is a conundrum. Burnout has multiple meanings and has been used to describe a range of phenomena from job frustration to suicidal depression. A meta‐analysis by Rotenstein et al.^6^ illustrated the disparate understandings of burnout. This analysis included 182 studies, wherein 142 different definitions of burnout were used. We characterize burnout similar to Leiter and Maslach^7^ as a syndrome of emotional exhaustion, depersonalization, and inefficacy.

Given the complexity and impact of burnout, identifying it poses a special and important challenge. Beginning the process of identification early in physician training is critical.^8^ For residency leadership, assessment of clinical knowledge and skills is intrinsic to evaluating resident performance. Potential signals for concern such as below‐average in‐training examination (ITE) scores have the potential to serve as markers for burnout risk. Further, enhancing our understanding as to when physicians in residency are at highest risk for burnout may allow for targeted interventions. This study aims to answer two questions: 1) does burnout risk correlate with examination scores and 2) does burnout risk change by postgraduate year (PGY) level?

### Importance

Prior efforts to define the association between clinical examination scores and burnout have shown conflicting results. A small‐scale study of 40 surgical residents with a response rate of 77% used the 22‐item Maslach Burnout Inventory (MBI) and the surgery ITE along with USMLE Step 1 and Step 2 results and found no correlation between burnout and examination scores;^9^ a similar study of 157 surgical residents with a response rate of 48% showed a correlation between lower scores and higher burnout rates when using the Oldenburg Burnout Inventory,^10^ and an orthopedic study of 62 residents with a 100% response rate using the MBI but “controlling for test‐taking ability” showed higher burnout rates correlated with lower scores. Only one unpublished study of 10 residency programs has assessed the association between ITE scores and burnout in EM residents.^11^

Similarly, prior studies have demonstrated conflicting data around the relationship between burnout and PGY level. Multiple previous studies have shown higher burnout rates in lower PGY levels.^12^, ^13^, ^14^ Others, including a study of burnout in EM residents, showed either no correlation with PGY level or increased rates in the PGY‐2 and/or PGY‐3 year alone, depending on the burnout definition.^8^, ^9^ Further defining the longitudinal course of burnout throughout residency training is needed and may assist in identifying effective interventions.

### Goals of This Investigation

This study was undertaken to determine the relationship between burnout risk in EM residents and performance on the American Board of Emergency Medicine (ABEM) ITE, as well as the potential changes in prevalence of burnout risk over the course of training.

## METHODS

### Study Design and Setting

This was a prospective, mixed‐methods, cross‐sectional cohort study to determine the association between burnout indicators and examination performance among EM residents. For burnout indicators, two MBI items derived from the MBI, a validated standardized inventory for the identification of burnout that consists of a 22‐item screening tool using a 7‐point Likert scale based on the frequency of feelings related to burnout, were used. These two items were used as described in prior studies.^15^, ^16^, ^17^ The functional understanding of burnout as defined by the MBI was the primary connotation adopted by the investigators.

The ABEM ITE is a multiple‐choice question examination taken by EM residents to prepare for the ABEM Qualifying Examination (QE) and indicates the likelihood that a resident will pass the written QE. The ITE assesses complex cognitive skills (e.g., diagnostic processing, clinical synthesis) as well as medical knowledge. To become certified by ABEM, a physician must first pass the QE followed by an oral certification examination.

The 2019 ITE was administered over a 5‐day period from February 26, 2019, to March 2, 2019. The test was administered in a proctored environment using a remote, secure, ITS (Internet Testing Systems, Baltimore, MD) testing platform. At the end of the ITE, residents were invited to complete a voluntary survey to assess their test experience. ABEM has provided postexamination surveys for over 20 years on previous tests and introduced the ITE survey in 2016. For the 2019 survey, ABEM added two items from the MBI that have been found to be strongly indicative of burnout as they capture the dimensions of emotional exhaustion and depersonalization: 1) one’s self‐perception of being burned out and 2) one’s feelings of callousness. Scoring high on the MBI scale for either of these dimensions is strongly indicative of an overall high burnout risk.^17^ Residents were asked to respond using a 7‐level Likert scale (1–7), ranging from very low frequency (1) to very high frequency (7). The intellectual property holder of the MBI limits the author’s ability to reproduce the MBI items. Interpreting scores is done cautiously because no single MBI score is “diagnostic” of a person being “burned out,” but rather may indicate the level of burnout risk. These two items have been successfully used by other investigators in lieu of the full MBI.^15^

In‐training examination question responses and survey results were initially stored in the ITS secure test administration platform and then transferred to secure data storage at ABEM. Survey response data and final scored test results were linked using a unique numeric identifier. Subsequently, all data were reported as aggregate, deidentified data. This study was reviewed and determined to be exempt research by the Mount Sinai Hospital Institutional Review Board (New York, NY).

### Selection of Participants

Residents in U.S. categorical EM residents who took the 2019 ITE were included. Residents who were excluded from analysis were those residents in international programs and residents in combined training programs.

### Interventions

There were no interventions.

### Measurements

Measurements included the number of residents in each year‐level of training (EM1–EM4), the two MBI item ratings, and the ABEM ITE score. Residents at a given level of training who were in EM1–3 training formats and EM1–4 were treated similarly, given the pre hoc assumption that the actual time in training has a larger effect on burnout risk than temporal progress within a particular program format. ITE performance is reported as an equated, scaled score between 0 and 100. Scaling and equating the scores provides more stable year‐to‐year performance comparisons that accommodates differences in the difficulty of the ITE from 1 year to the next.

### Outcomes

There were three outcomes: ITE scores and the responses to the two MBI items.

### Data Analysis

Likert responses to each MBI item were determined for each training level under the a priori assumption that the frequency of responses indicating a greater risk of burnout would increase during training. Performance, as measured by the scaled, equated score, was compared to the MBI item responses. A corrected Spearman’s correlation coefficient (ρ) was used to compare continuous data (score) against a discrete ordinal variable (MBI Likert response). Due to the size of the sample, significance was determined a priori to be α < 0.01. All analyses were performed using R statistical software (version 3.6.0) and the *haven* and *readxl* packages (R Foundation for Statistical Computing).

## RESULTS

There were 8,043 physicians who took the ABEM ITE, with 7,712 test takers (95.9%) meeting the definition of the study group. There were 2,501 EM1 residents, 2,389 EM2 residents, 2,206 EM3 residents, and 616 EM4 residents in the study group. There were 7,206 (93.4%) physicians who completed the first MBI question about burnout (Table 1); 7,172 (93%) completed the second MBI question about callousness (Table 2).

**Table 1:** MBI Burnout Item

Frequency	EM1	EM2	EM3	EM4	Total
1 (very low frequency)	141 (6.0)	105 (4.7)	96 (4.7)	19 (3.3)	361 (5.0)
2	478 (20.2)	437 (19.5)	438 (21.6)	111 (19.5)	1,464 (20.3)
3	514 (21.7)	399 (17.8)	332 (16.4)	105 (18.4)	1,350 (18.7)
4	697 (29.4)	645 (28.8)	509 (25.1)	154 (27.0)	2,005 (27.8)
5	221 (9.3)	211 (9.4)	220 (10.8)	55 (9.6)	707 (9.8)
6	249 (10.5)	341 (15.2)	321 (15.8)	97 (17.0)	1,008 (14.0)
7 (very high frequency)	67 (2.8)	103 (4.6)	112 (5.5)	29 (5.1)	311 (4.3)

Data are reported as *n* (%).

MBI = Maslach Burnout Inventory.

**Table 2:** MBI Callousness Item

Frequency	EM1	EM2	EM3	EM4	Total
1 (very low frequency)	363 (15.4)	220 (9.9)	179 (8.9)	38 (6.7)	800 (11.2)
2	627 (26.6)	496 (22.3)	455 (22.6)	109 (19.2)	1,687 (23.5)
3	459 (19.4)	359 (16.1)	307 (15.2)	88 (15.5)	1,213 (16.9)
4	449 (19.0)	473 (21.2)	406 (20.1)	128 (22.5)	1,456 (20.3)
5	172 (7.3)	221 (9.9)	188 (9.3)	57 (10.0)	638 (8.9)
6	214 (9.1)	338 (15.2)	317 (15.7)	89 (15.6)	958 (13.4)
7 (very high frequency)	76 (3.2)	121 (5.4)	163 (8.1)	60 (10.5)	420 (5.9)

Data are reported as *n* (%).

MBI = Maslach Burnout Inventory.

The average ITE score across all training levels was 71.7 (95% confidence interval = 71.6 to 71.9). There was no statistically significant association between the MBI burnout item response and ITE performance (ρ = −0.03; p = 0.015). There was a positive, statistically significant association between the MBI callousness item response and higher ITE performance (ρ = 0.07; p < 0.001). Although this relationship was statistically significant, the magnitude of the relationship was very small. The level of training more substantially correlated with ITE performance (ρ = 0.46; p < 0.001).

**Figure 1:**
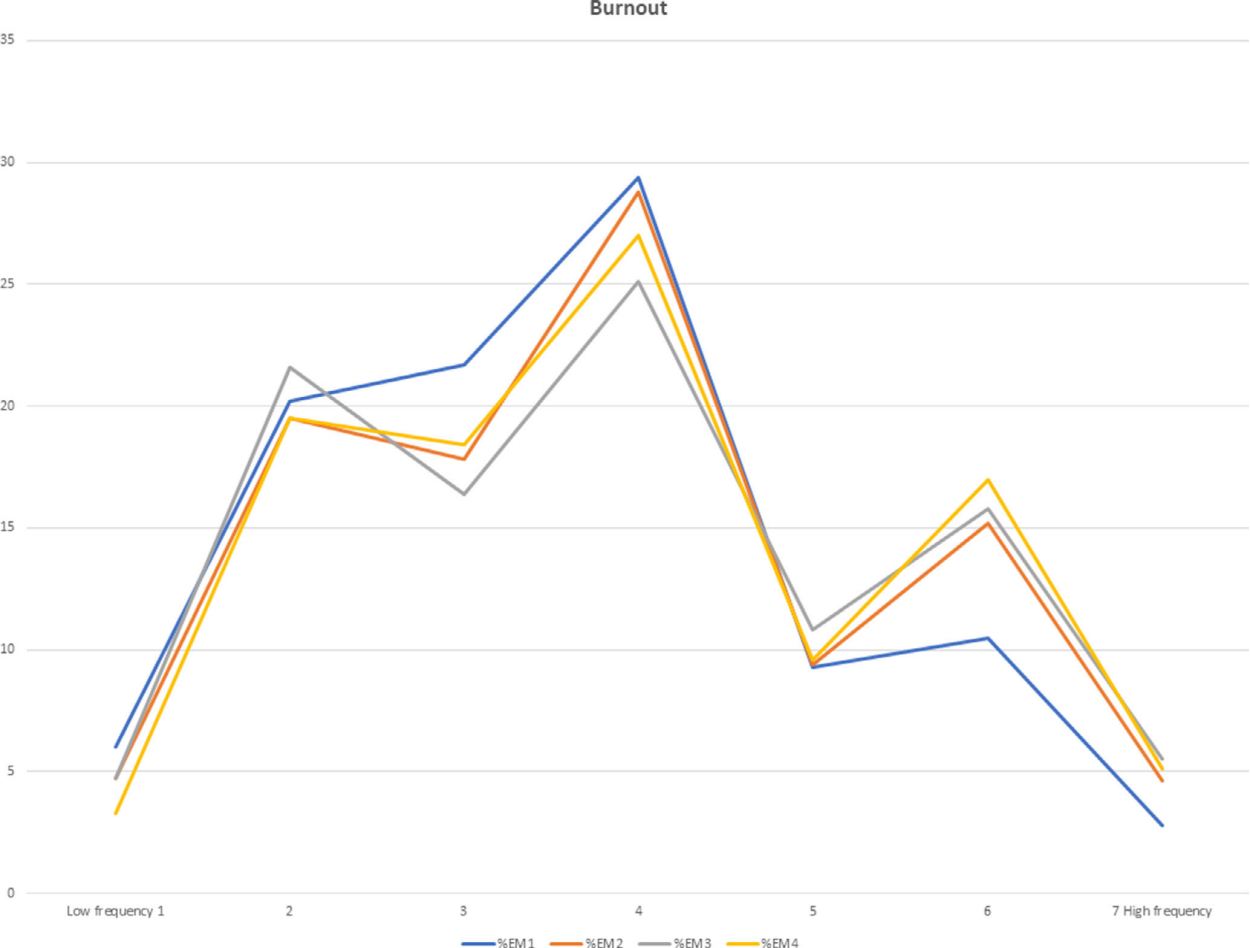
Burnout item responses by PGY level.

**Figure 2:**
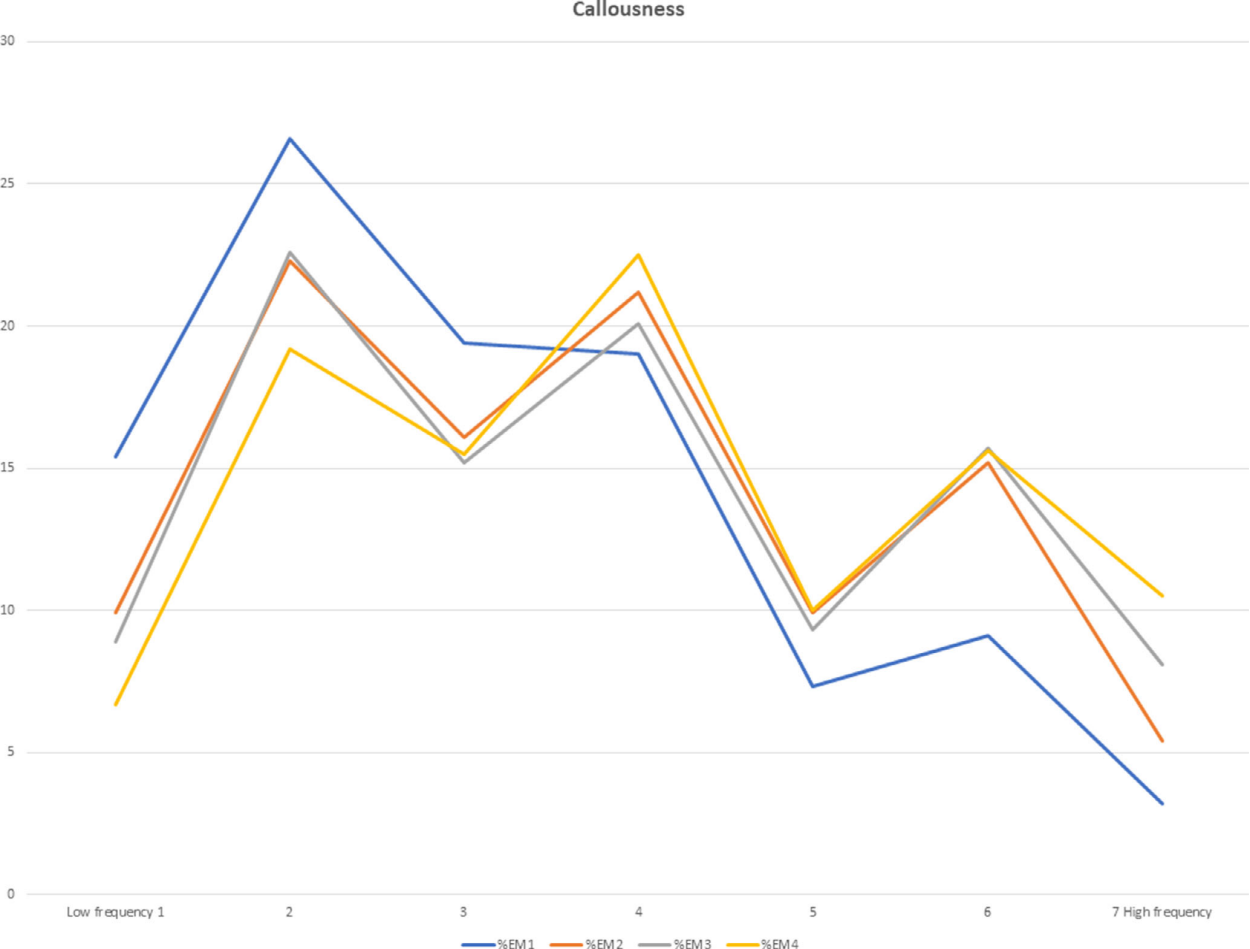
Callousness item responses by PGY level.

A greater percentage of residents at a higher training level reported a greater frequency of burnout self‐perceptions (Table 1, Figure 1). More experienced residents also reported a greater frequency of callousness (Table 2, Figure 2). There was a statistically significant association between the response to the burnout self‐perception item and training level (ρ = 0.07; p < 0.001); however, the magnitude of the relationship was small. There was also a statistically significant association between the response to the callousness item and training level (ρ = 0.15; p < 0.001). The magnitude of this relationship was also small. In combination, high scores on both items are indicative of an overall increased burnout risk for those further along in training.^15^, ^16^, ^17^, ^18^ A high score (≥5) is indicated by the feeling being present at least weekly.

An earlier study by Dyrbye et al.^17^, ^18^ used the two MBI items to determine the prevalence of burnout in residents. The prevalence derived from this analytic approach should be interpreted cautiously since the MBI manual cautions against the use of a single cutoff level to determine a dichotomous result. However, when this approach was applied post hoc to the data in our study, the results showed the prevalence of burnout risk to increase with training level (2 × 4 chi‐square test, p < 0.001).^17^ Using the Dyrbye dichotomy criteria for both MBI items in combination, with a cutoff of 5 on the Likert scale indicating high overall burnout risk, the prevalence of burnout risk in various training levels were EM1, 28.2%; EM2, 39.0%; EM3, 41.1%; and EM4, 43.3%.^17^

## DISCUSSION

Burnout among EPs is an area of serious concern for the specialty. This study is the first large‐scale study to examine the prevalence of burnout indicators and their association with examination performance and PGY level for EM residents.

Burnout is a complex syndrome that both the 22‐item MBI and its shortened counterparts are not designed to diagnose. Indeed, in the original applications of the MBI, Maslach noted that it is “usually used to assess a group of staff members in an organization rather than as an individual diagnostic instrument.”^19^ As such, for the purposes of this study, we consider the MBI useful in assessing high, medium, or low *risk of burnout* among members of a group of EPs, rather than being diagnostic of the burnout syndrome. When using the two‐item MBI as opposed to the full 22‐item MBI for the purpose of screening a large sample of EPs, responses to the burnout item serve as surrogates for burnout risk in the emotional exhaustion domain while responses to the callousness item are indicative of risk of burnout in the depersonalization domain, both with strong positive predictive values for burnout.^15^, ^16^ In combination, these two items are highly predictive of overall burnout risk.^8^, ^15^, ^16^, ^17^, ^18^, ^19^ Nonetheless, the two‐item MBI is not intended for use in monitoring or diagnosing burnout in individuals, but rather for use in risk stratification for burnout in sample populations.

Although the association was weak, our study showed an increase in overall burnout risk with a longer period of training when responses to the two MBI items were combined. Prior studies have demonstrated reduced levels of compassion as trainees progress through residency, which may coincide with our findings, particularly when considering the dimension of depersonalization (callousness).^20^ An EM residency–based study with a survey response rate of 21.2% showed higher burnout rates in PGY2 and/or PGY3 year or no correlation with PGY level depending on metrics used.^8^ For EM residents, clinical uncertainty, lack of autonomy, and clerical duties are major contributing factors to burnout.^21^ Other studies have identified contributors such as electronic health record use,^22^ racial bias, ^17^ gender,^23^ and duty hours violations.^24^ Practicing EPs have also identified factors that contribute to their burnout including work hours, shift work, nonclinical duties, and clerical tasks. As residents progress through training, it is possible that contributors to burnout shift in a similar fashion and, as such, identifying when residents are experiencing burnout is important for timely intervention. Further, our study demonstrated a significant level of burnout risk for all EM residents surveyed, regardless of training level, which may have concerning implications. Burnout has been associated with depression and increased thoughts of suicidality,^23^ reduced patient safety,^25^ and negative overall program culture.^24^ Each of these factors also contribute directly to the quality of patient care and interaction.

Prior studies looking at burnout and examination scores have mixed results with several finding that increased levels of burnout were associated with worse performances on the ITE. This result is counter to our findings, which did not find a statistically significant association between feeling burned out and ITE performance. Further, our findings indicated a direct relationship between callousness and examination performance.

The implications of our findings may indicate resilience of EM residents in the face of burnout risk factors. The ability to consolidate knowledge and interpret complex medical scenarios in a testing environment may also confer a separate skill less impacted by burnout than clinical work with patients, which has been shown to correlate strongly with burnout. Additionally, regarding our findings with respect to callousness, other interpretations could be that callousness may be protective or may confer better test‐taking skills or perhaps that better test takers are more prone to callousness. Although poor performance on the ITE may be a marker of an unknown stressor in a resident, our study does not support concern for increased burnout markers relative to ITE performance. Instead, initiatives for decreasing burnout may result in decreased suicidality thoughts,^3^ decreased medical errors,^4^ and an overall greater satisfaction of medicine as a career.^5^ These results may translate to improved quality of patient care and interactions, but not necessarily to improved ITE scores.

As a practical matter, these findings suggest that self‐reported burnout indicators by EM residents do not have a substantial relationship with ITE performance. This may have important implications for monitoring burnout risk. That is, for residency program leadership seeking measures and “red flags” that may indicate whether residents are burnt out, the ITE may have limited use as a monitoring device. Specifically, those perform well on the ITE may still be at high risk for burnout and those who score poorly on the ITE may have important reasons outside of burnout for doing so.

There is a keen opportunity for further research in this area. It would be important to study the longitudinal trend in burnout indicators and contributors while physicians are undergoing training. Another study could compare the ITE performance between physicians with the highest risk for burnout and lowest risk for burnout. Finally, understanding those factors during training that contribute to burnout as well as effective remedies would contribute to better understanding a vexing problem.

## LIMITATIONS

This study has several limitations. First, the survey data were self‐reported responses and there was no attempt to verify the veracity of the responses.

Second, this study used a cross‐sectional, not a longitudinal, design. Given this design, the authors cannot make any causal statement about the acquisition or development of burnout or callousness during residency training. A longitudinal model would determine the risk of burnout as residents advance through training.

Third, although there were statistically significant associations between training level and the MBI items, in part due to the large sample size, the magnitude of the association was relatively small. Fourth, no operational definition of burnout was provided for survey respondents. Survey takers were required to self‐define their understandings of the burnout and callousness items which allows for variable interpretations. However, as demonstrated and verified in prior studies, scoring high on the MBI scale for both of these dimensions is still strongly indicative of an overall high burnout risk, and assessing scores for each individual item strongly correlates with the emotional exhaustion and depersonalization dimensions of the full MBI.

Fifth, given the inability of the two MBI items to define who is “burned out,” instead only defining who may be at risk for burnout, this study is unable to definitively determine the prevalence of burnout among EM residents. Additionally, we used a two‐item MBI rather than the comprehensive 22‐item MBI, which limits the validity of the data. However, the results can be compared to other studies that used similar methods and definitions of burnout.^15^, ^16^, ^17^, ^26^

Sixth, this survey was administered upon completion of the ITE. Stress related to the examination itself as well as the potential relief upon completion of the examination could influence the burnout perception at the time. However, the frequency‐based nature of the MBI scale is intended to control for this potential confounding factor.

Seventh, although the survey was assured to be confidential, social desirability bias may have affected results. That is, there may have been individual resident concerns over the potential nonconfidentiality of the survey responses, given that the examination results are identified. This may have caused underreporting of burnout survey responses. However, the authors have no way of determining any impact of this potential bias on the results.

Finally, just as the results of other specialties’ studies around burnout cannot be confidently generalized to EM, results of this study cannot be generalized to other specialties. EM has been identified as a specialty that is associated with one of the highest rates of burnout with unique contributing factors. Additionally, our study examined the correlation between burnout and test performance; applying these findings to any impact on clinical practice would be inappropriate.

## CONCLUSION

Our study indicates that residents at higher levels of training reported stronger self‐perceptions of burnout risk in both the emotional exhaustion and the depersonalization domains. Both Maslach Burnout Inventory items had statistically significant associations with training level, although weak. Our study showed that the correlation between self‐perception of burnout and in‐training examination performance was not statistically significant. The correlation between feelings of callousness and in‐training examination performance had positive statistical significance, although weak. Our study indicates little relationship between the overall risk of burnout and in‐training examination performance based on these two indicators.
